# Our Dumb Animals

**Published:** 1875-04

**Authors:** Geo. G. Lobdell


### OUR DUMB ANIMALS.

An intelligent subscriber to this journal, Mr. George G. Lobdell, President of the Lobdell Car-Wheel Company, of Wilmington, Delaware, observed our recent request for particulars concerning the treatment of diseases of our dumb servants, and communicates to us his method of controlling a very dangerous malady from which horses have recently suffered in various parts of the country. This gentleman is an extensive horse-breeder, and his statements are those of a very intelligent and competent gentleman. His practice is certainly rational and excellent. He says:—

I herewith give you a description of my method of treating a peculiar nervous influenza, known as *spinal meningitis*, which has widely prevailed of late.

The symptoms which characterize this disease, so far as my observations extend, and which have been confined to my own horses, are, loss of appetite, dullness, more or less debility, and great weakness, which is manifest more particularly in the posterior extremities,—due, I think, to partial paralysis; pulse quick, from 40 to 45, but generally weak; respiration accelerated.

Sometimes they are taken with a chill, and sometimes very marked indications of inflammation of the lungs (pneumonia) are soon manifest. In some there is a slight soreness of the throat, accompanied also with a slight cough, and light discharges from the nose. The attacks have always been sudden, and without any apparent premonitory symptoms. The animal will appear perfectly well at night and be found sick in the morning. Some appear more affected across the loins (partially paralyzed) than others.

Those of my horses which became sick first, were taken during my absence from home, and were given medicine and rubbed with mustard. Those that got medicine and recovered were much longer in getting well than those that did not; and one, the Hambletonian stallion Harbinger, died. All that have been taken since (twelve) have been treated with perfect success with the hydropathic or wet pack, which I apply as follows:—

I use an old bed-quilt that will completely envelope the breast and body; cut in it two slits, so that it will fit around the fore legs when placed under the body, so that it will lap over on the back. Around this I put common horse-blankets, the whole secured well by a plow-line or surcingles, the intention being to envelope the whole body, breast, and lower part of the neck, closely and completely with the wet quilt. If the throat is affected, I put an additional pack around the throat or have the quilt long enough to cover the throat. The quilt should be taken off and rinsed every three hours, the animal washed thoroughly with water, not warm, but of a temperature suited to the degree of fever, so as not to produce chilliness; then the animal thoroughly rubbed with straw or a cloth, and the pack renewed. The pack I usually wet with water of a temperature such as used by women in washing, but not so wet as to drip.

My horses have not been disposed to lie down until they begin to get better. As soon as I discover the first symptoms of sickness I put them in a pack and continue it, with or without intermission, as the violence of the attack indicates is necessary, or until the pulse and breathing are better. If not very bad, I use the wet pack during the day only, and a dry pack at night. I use great care in feeding, until they are entirely recovered; give scalded bran and oats in small quantities, and all the water they will drink, but no medicine whatever.

This disease can not be attributed to want of proper food or care; for most frequently it attacks horses in the best condition, and those that have been treated with great care. I shall not attempt to assign a cause for it. It may be atmospherical, or due to a blood poison; but from the great success which has followed this treatment, as well as from the symptoms, I believe that it is inflammatory in its nature. I have used the pack with like success in inflammation of the pleura, inflammation of the lungs (pneumonia), founder, and inflammatory rheumatism. I have been asked, do horses have inflammatory rheumatism? I answer, yes. I have treated as clear a case as was ever manifest in a human being. I believe the treatment beneficial in all diseases arising from blood poison or indigestion.

Those of my horses that have been packed—giving them one pack each day for two or three days—have not had the disease.

GEO. G. LOBDELL.

---

It should be made honorable for men to live according to their means, and dishonorable to live beyond their means.

